# Interactive Clinical Avatar Use in Pharmacist Preregistration Training: Design and Review

**DOI:** 10.2196/17146

**Published:** 2020-11-06

**Authors:** Jessica Thompson, Simon White, Stephen Chapman

**Affiliations:** 1 School of Pharmacy and Bioengineering Keele University Keele United Kingdom

**Keywords:** virtual patient, high-fidelity simulation training, patient simulation, pharmacy education, pharmacy practice education, virtual reality

## Abstract

**Background:**

Virtual patients are interactive computer-based simulations that are being increasingly used in modern health care education. They have been identified as tools that can provide experiential learning and assessment in a standardized and safe environment. However, the study of high-fidelity virtual patients such as interactive clinical avatars within pharmacy is limited.

**Objective:**

The aim of this paper is to describe the design and review of three interactive clinical avatar simulations as part of pharmacist preregistration training.

**Methods:**

A multistep design approach was taken to create interactive clinical avatar simulations on the topics of emergency hormonal contraception (EHC), calculation of renal function, and childhood illnesses. All case studies were reviewed by registered pharmacists to establish content and face validity. The EHC case study and data collection questionnaire were also reviewed by a purposive sample of preregistration trainees and newly qualified pharmacists. The questionnaire used Likert ranking statements and open-ended questions to obtain users’ feedback on the design, usability, and usefulness of the interactive clinical avatars as learning tools. Descriptive statistics and content analysis were undertaken on the data.

**Results:**

Ten preregistration trainees and newly qualified pharmacists reviewed the EHC interactive clinical avatars and data collection questionnaire. The data collection questionnaire was associated with a Cronbach alpha=.95, demonstrating good reliability. All three interactive clinical avatar simulations were reported as usable and appropriately designed for preregistration training. Users perceived they were developing skills and knowledge from the simulations. The high-fidelity nature of the avatars and relevance of the simulations to real-life practice were reported as aspects that encouraged the application of theory to practice. Improvements were suggested to ensure the simulations were more user-friendly.

**Conclusions:**

The design and creation of the interactive clinical avatar simulations was successful. The multistep review process ensured validity and reliability of the simulations and data collection questionnaire. The in-depth explanation of the design process and provision of a questionnaire may help widen the use and evaluation of interactive clinical avatars or other simulation tools in pharmacy education. The interactive clinical avatars were reported as novel learning tools that promoted experiential learning and allowed users to feel like they were engaging in real-life scenarios, thus developing transferable knowledge and skills. This may be potentially beneficial for many health care training courses as a way to provide standardized experiences promoting active learning and reflection.

## Introduction

Virtual patients are defined as “a specific type of computer-based program that simulates real-life clinical scenarios [in which] learners emulate the roles of health care providers to obtain a history, conduct a physical exam, and make diagnostic and therapeutic decisions” [1]. Virtual patient is an umbrella term that includes computer-based tools such as still photos, video clips, avatars, and immersive virtual reality simulations. The key element for a tool to be described as a virtual patient is that the simulation outcome must be dependent on user input, their prime differentiation from other e-learning tools [2].

An interactive clinical avatar is classified as a high-fidelity form of virtual patient [3]. The avatar is a computer-generated, 3-dimensional (3D) animation that represents a patient or health care professional. Avatars can be either asynchronous, accessed anytime and providing responses without the requirement for a tutor online, or synchronous, active in real time and requiring input from multiple users or educators [4,5].

Interactive clinical avatars have been evaluated less frequently in pharmacy education and training than in other health care professions and less frequently than other forms of virtual patients [2,6,7]. This may be as a result of potential barriers associated with their production and use including information technology skills, cost, and time constraints [6,8,9]. Evaluations of asynchronous avatars in pharmacy education have demonstrated significant positive outcomes in the development of students’ knowledge, skills, and confidence to interact with real patients [10-15]. The majority of this research has used self-reporting scales based on individuals’ perceptions to evaluate outcomes. The drawbacks of such scales is their inherent subjectivity; only individuals’ perceptions can be reported, with the appropriateness of statistical analysis to determine significance greatly debated in the literature [16,17].

Pharmacy preregistration training in the United Kingdom is a workplace-based training year predominantly occurring in a community or hospital pharmacy. Performance standards must be met during the preregistration training year before trainees can take the preregistration examination and be registered as a pharmacist. Experiences of preregistration trainees can vary considerably and are dependent on multiple factors including sector of training (hospital vs community), training site, tutor support, and individual initiative [18,19]. Notable differences in the quality of preregistration training have resulted in a disparity in preregistration examination pass rates between the sectors of training [20]. The role of a pharmacist in the United Kingdom is evolving, and training needs to adapt to ensure all individuals qualify with appropriate knowledge and skills to provide safe and effective patient care [21].

Simulation has the potential to reduce variation in preregistration training by providing standardized experiences, thus ensuring a more controlled development of trainee competence. Miller’s triangle is the most commonly used model to measure competency development in pharmacist preregistration training [22]. A benefit of simulation, especially high-fidelity simulation, is the promotion of experiential learning, which aids user progression to the “shows how” level of competence, initially proposed as difficult to reach without real-world experience [23,24]. Immersive simulations provide a safe environment for individuals to practice specific skills or knowledge without risk of harm to a real patient [25]. Evaluation of high-fidelity simulations is essential moving forward with the increasing numbers of pharmacy students entering university, changing role of a pharmacist, and problems with standardizing placements [26]. Experience alone may not be enough for individuals to obtain mastery of clinical skills [27], and simulation-based learning can be instrumental in promoting experiential, situated learning and bridging the gap between theory and practice without differences in educational outcomes [28].

Only one study has evaluated interactive clinical avatars in pharmacist preregistration training, leading to the creation of 3 interactive clinical avatar case studies covering different competencies [10]. The interactive clinical avatar software used has 3 key parts associated with and essential to its design: an electronic database containing the avatar’s responses, a computer-generated graphic, and a system to link the two together [29]. The brain of the avatar is the electronic database based on a modified Markov model design [30], which uses a decision tree to map the progress of a case. As a user progresses through a case, the database monitors the path through the decision tree and collates positive and negative feedback to be given at the end of the simulation [31]. The body of the avatar is a computer-generated graphic of a 3D character. The final part of the system is the heart, which carries information from the brain to the body, allowing a real-time, immediate response.

The aim of this paper is to describe the design and review of three interactive clinical avatar simulations that are part of pharmacist preregistration training.

## Methods

### Intervention Development

A multistep design approach was taken.

#### Case Design

Case studies were designed and created by the research and digital development teams at Keele University School of Pharmacy and Bioengineering from a review of the literature, the preregistration syllabus, and discussions between the lead researcher (JT) and first year qualified pharmacists who had recently completed their training year. Interactive clinical avatar simulations were created on the topics of emergency hormonal contraception (EHC), calculation of renal function, and childhood illnesses to develop a range of knowledge and skills essential for preregistration training and future practice. Due to training variations, trainees may find it difficult to demonstrate competence in these areas. The clinical elements of the cases were based on appropriate guidelines and resources that pharmacists use in everyday practice.

The simulation on EHC supply was created because trainees completing their preregistration training in a hospital would be less likely to see or be actively involved in an EHC consultation than those in the community sector. Individuals training in a community pharmacy are only able to observe a consultation, so a simulation was created to provide an opportunity to actively advise on the use of EHC. The calculation of renal function was incorporated into a simulation because hospital pharmacists calculate patients’ renal function and adjust drug doses more often than those working in community pharmacy. The childhood illness case study was designed around measles because of its increasing prevalence in the United Kingdom and the need for pharmacists in the community and hospital sectors to have the knowledge to distinguish it from other conditions [32].

#### Script Design

**Figure 1:**
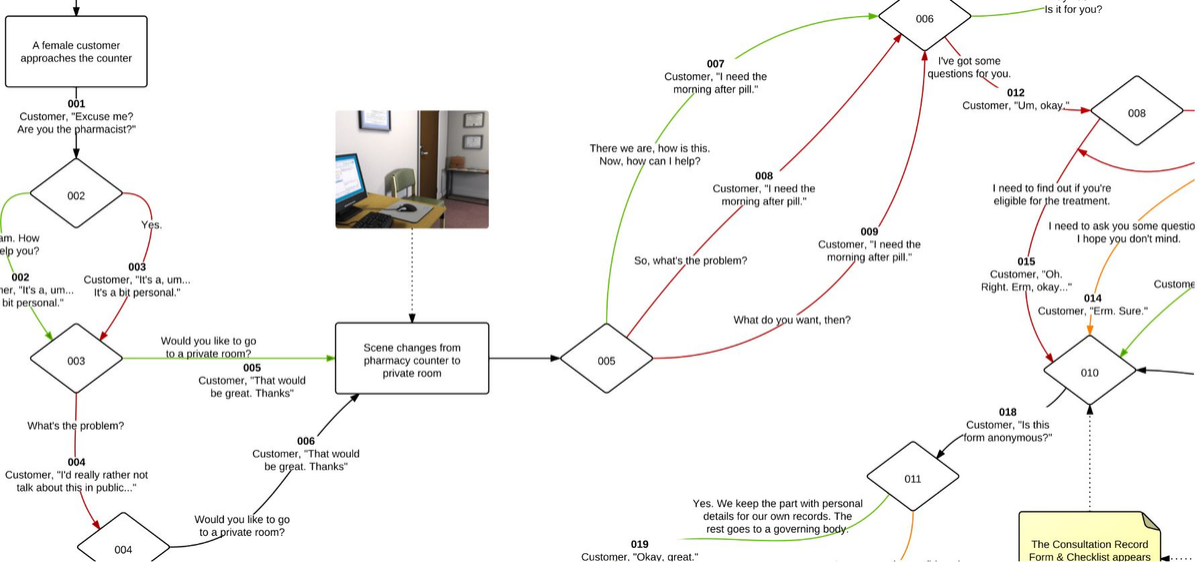
Markov model decision tree for the emergency hormonal contraception simulation.

The scripts for the interactive clinical avatar cases were designed to meet predefined learning objectives and ensure equivalent user experiences. The research team worked in consultation with a community pharmacist on the EHC case and a hospital pharmacist in the renal function case. The scripts were reviewed and amended according to the principles of constructive alignment in face-to-face meetings and through email until realistic and accurate simulations were created that aligned with the intended learning outcomes [33]. A decision tree of the script for each case was developed that established key points and decisions. Each case had an ideal pathway, and decisions were categorized as more or less favorable compared with this (Figure 1).

Different script styles were created depending on the intended learning outcomes of the simulation. Standard navigation through virtual patient simulations is via predefined menu inputs [34]; however, different inputs were used in the design to widen the scope of the interactive clinical avatars used and evaluate user preferences in a larger study [35]. Multiple-choice inputs were selected for communication skill development, whereas free-text inputs were used to encourage knowledge development [36-38]. The interactive clinical avatars were designed to provide feedback to users. This feedback, spoken by the avatar and provided in textual format, specifically laid out what a user had done well and how they could improve. This provision was selected to encourage a reflective approach to learning, as was the ability for a user to repeat the simulation and change their path to receive a different patient outcome and feedback.

#### Avatar Design

The scripts and avatars were designed to be realistic but not to the detriment of falling into the uncanny valley as described by Mori [39], who suggested that objects portraying human characteristics may be viewed positively up to a certain point beyond which the degree of visual similarity to real humans becomes unsettling and can trigger negative thoughts [40-42]. All avatars were designed to express humanistic characteristics through simple body language, movements, and prerecorded voice replies. As an example, the avatar in the EHC simulation was animated to blush when asking for the morning after pill, as may happen in real practice due to the sensitive nature of the topic.

The simulations were stored online and created using standard HTML. Javascript functions were used to handle page logic, including processing button clicks for both the free-text input and multiple-choice questions. For the interactive clinical avatar simulations using free-text inputs, once the Speak to Patient button was clicked, the text was sent to a web service hosted on the same server to process the text. This returned a code to the site processed on the client side via JavaScript (using the jQuery library) to determine what animation to play and how the case should continue. When multiple-choice inputs were used, processing occurred client side; skipping the web service step. The avatars were modeled, textured, and animated using Maya 3D software (Autodesk Inc). Still images were rendered using mental ray (Autodesk Inc) and then composited using After Effects (Adobe) to create the final MP4 animation files. The avatars’ verbal responses were audio recordings of a voice actor who was chosen to match the avatars’ characteristics.

**Figure 2:**
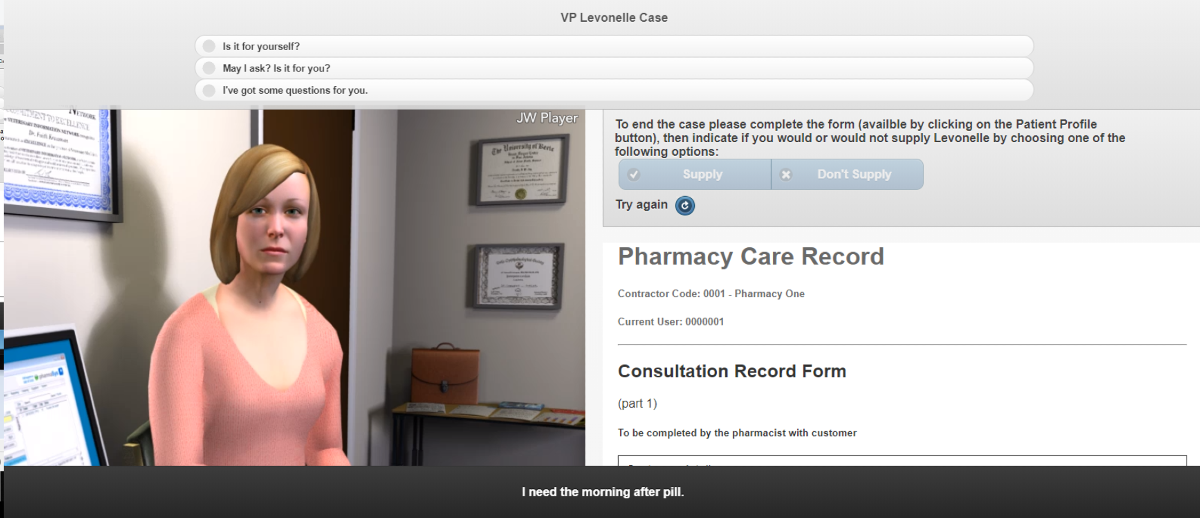
Screenshot of the emergency hormonal contraception avatar simulation.

The avatar in the EHC simulation was a young woman who presented to a community pharmacy requesting the morning after pill (Figure 2). The user had the option to invite the avatar into a consultation room to ensure a private, confidential conversation, and the simulation background would change to show this. The simulation used a mix of multiple-choice and free-text inputs. To aid the consultation and mirror real life, a consultation form was built into the simulation and included as a separate tab, which could be opened and completed by the user as they progressed through the consultation to help them make a decision on the appropriateness of EHC supply.

**Figure 3:**
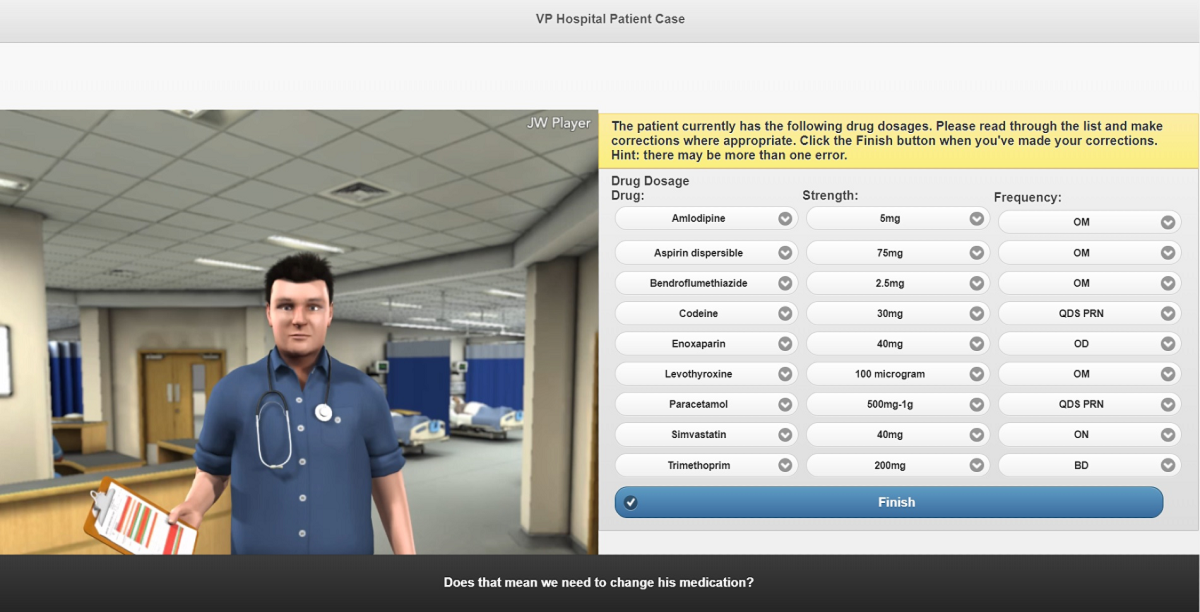
Screenshot of the renal function avatar simulation.

In the renal function simulation, users played the role of a hospital pharmacist and interacted with an avatar of a doctor on a hospital ward via multiple-choice input (Figure 3). The simulation integrated hospital notes and a drug chart that could be accessed throughout the simulation and contained the same sort of information as would be expected in a real set of hospital notes, including test results and medication history. Users were required to calculate the patients’ renal function using the information provided and adjust drug doses accordingly to help improve their calculation and clinical skills.

**Figure 4:**
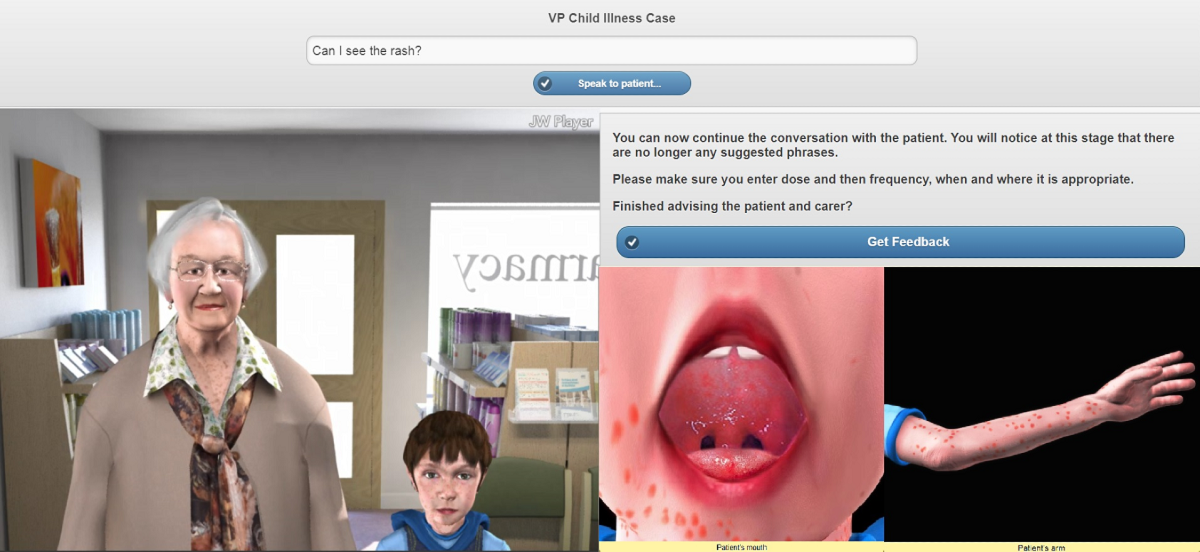
Screenshot of the childhood illness avatar simulation.

The childhood illness simulation used two avatars: a grandmother and her grandson (Figure 4). The simulation was set in a community pharmacy, and users interacted with both avatars via free-text input. The grandson avatar presented with measles. When users asked to look at the rash, an enlarged image of the child’s arm appeared showing a more detailed image of the rash associated with measles; similarly, if the user requested to look inside the child’s mouth, an enlarged image of the oral cavity appeared showing Koplik spots. These images remained available throughout the simulation and demonstrated the high-level fidelity of the technology. The user was directed to make a diagnosis, and upon success, they were directed to recommend appropriate licensed medicines and self-care advice. Avatars were not based on real patients, and any resemblance to a person, living or dead, is coincidental.

A YouTube video has been created as a demonstration of the EHC avatar simulation [43] (Multimedia Appendix 1).

### Interactive Clinical Avatar Evaluation

#### Design

During the initial design phase, an internal review took place within the research team with advice from the digital development team and feedback from registered pharmacists. The interactive clinical avatar simulations were tested by pharmacists within the School of Pharmacy and Bioengineering at Keele University; all members of staff who were pharmacists were contacted, including those who had assisted with the initial script design. Links to the interactive clinical avatar simulations were sent via email, and pharmacists were asked to provide electronic or face-to-face feedback on any areas of the case studies, including clinical and pedagogical aspects.

In addition, the EHC case study and data collection questionnaire were reviewed by a sample of preregistration trainees and newly qualified pharmacists. Ethical approval was granted by the Keele University Ethical Review Panel.

#### Sampling and Recruitment

A purposive sample of 30 preregistration trainees and newly qualified pharmacists who were Keele University alumni (n=10) or members of the British Pharmaceutical Students Association (n=20) were invited to participate over social media. Consideration was given to the sample size based on the pool of available participants and the required sample size for the wider study [44]. Previous literature has identified a range of sample sizes for studies designed to review data collection materials, with a minimum of 10 established as appropriate to evaluate adequacy of instrumentation [45]. A range of views was sought for this evaluation, and thus recruitment included individuals who had studied at different universities, were in different stages of their education, and were currently completing or had completed their preregistration training in a community or a hospital pharmacy. The study used a sample that best represented those with knowledge of the research topic, in this case current preregistration trainees and individuals who had just finished preregistration training. Only one round of recruitment occurred, as saturation of themes was reached [46].

#### Data Collection

Participants were asked to complete the interactive clinical avatar case study on the knowledge and law of supplying EHC and a questionnaire to gather their feedback on the simulation and the data collection tool. Evaluative questionnaires are an established method to compare thoughts on different learning tools [47,48], but a review of the literature identified a lack of validated instruments to evaluate interactive clinical avatars as learning tools [49]. A data collection questionnaire was therefore created for this research by adapting questions from validated evaluation instruments found in the educational literature to provide a tool to evaluate perceptions of virtual patients in pharmacy education for future studies [50-52].

This questionnaire consisted of 20 Likert scale ranking statements (1= strongly disagree, 2= disagree, 3= undecided, 4= agree, 5= strongly agree) and a series of open-ended questions and was subject to measurements of reliability and validity. Review by the supervisory team, practicing pharmacists, preregistration trainees, and newly qualified pharmacists ensured its content, face validity, and internal consistency [53-55].

#### Data Analysis

Quantitative data from the Likert scale were analyzed descriptively. Qualitative data from the open-ended questions were subject to content analysis [56]. All comments provided by participants were imported into an Excel spreadsheet (Microsoft Corp) for coding. The content analysis process involved the identification, organization, and indexing of comments to provide frequencies of coded themes [57]. The lead researcher (JT) conducted the initial content analysis. These codes were reviewed by the research team and discussed to reach consensus, providing researcher triangulation. Cronbach coefficient alpha measured internal reliability of the data collection tool [58].

## Results

### Demographic Results

Of 30 invited, 10 participants responded and reviewed the EHC interactive clinical avatar and data collection questionnaire: 4 were Keele University alumni and 6 studied at other universities and were members of the British Pharmaceutical Students Association. Four of the participants were preregistration trainees, and 6 were first-year qualified pharmacists; 7 of the participants were female and 3 were male. All participants were aged 20 to 25 years. Participants were of White British (7/10), Indian (1/10) and Chinese (2/10) ethnicities. An equal number of participants were from hospital and community practice. A greater proportion of participants (6/10) reported no previous experience with interactive clinical avatar simulations.

### Questionnaire Validation

Initial reliability testing of the instrument consisted of establishing content and face validity. The research team as pharmacists had first-hand knowledge of the construct of interest from practice, and they could advise on the questionnaire content and language. The review of the questionnaire by three qualified pharmacists contributed to addressing the content validity of the instrument. This resulted in minor typographical changes, but the questionnaire was otherwise reported as appropriate to measure user views on interactive clinical avatar simulations.

All preregistration pharmacists and newly qualified pharmacists reported the questions and Likert statements to be clearly written and unambiguous, further confirming internal validity of the data collection tool. Cronbach α=.95, which demonstrated a high level of internal consistency suggesting that the Likert scales were reliable to be used in a bigger study [44].

### Quantitative Analysis

The statements from the Likert ranking scales, their descriptions, and the associated median agreement scores are shown in Table 1. For all Likert ranking statements, participant median agreement scores were in the agree or strongly agree categories. Fewer participants ranked themselves as undecided, and only on 5 occasions did participants disagree with a statement. No participants ranked themselves as a 1 (strongly disagree) for any statement.

The fidelity of the simulation was reported as realistic by 80% (8/10) of participants, with it making them feel as if they were the pharmacist and were making the same decisions as in real practice (70% [7/10] and 90% [9/10] agreement, respectively). The simulation was reported as enjoyable (9/10, 90%), interesting (10/10, 100%), and set at the right level for preregistration training (9/10, 90%). The majority of participants reported that the simulation was easy to access (9/10, 90%) and the intended learning outcomes were easy to understand (7/10, 70%), indicating high usability as a learning tool.

The level and specificity of the feedback was reported as adequate for participants’ learning needs (10/10, 100%). The development of skills for future practice from the simulation was reported by 80% (8/10) of participants, with clinical reasoning skills being developed to a greater extent than problem-solving or decision-making skills (90% [9/10] vs 80% [8/10]). There was high agreement regarding the development of and application of knowledge from using the simulation (100% [10/10] and 80% [8/10], respectively). Participants reported feeling more confident in caring for patients (9/10, 90%), collaborating with other health care professionals (8/10, 80%) and practicing as a pharmacist (8/10, 80%) after completing the simulation.

There were some differences between data received from preregistration trainees and newly qualified pharmacists. Preregistration trainees agreed or strongly agreed with the majority of Likert statements; one participant (25%) was undecided with statements 2 and 3 relating to really feeling like the pharmacist in the simulation. Two newly qualified pharmacists (33%) also ranked themselves as undecided on statement 2. Newly qualified pharmacists were reportedly more undecided as to the simulation aiding the development of skills for practice (2/6, 33%) and allowing the application of theory to practice (2/6, 33%). There were a few statements on which newly qualified pharmacists disagreed: aiding the development of problem-solving and decision-making skills (1/6, 17%), individual responsibility to learning (1/6, 17%), collaborating with patients (1/6, 17%) and health care professionals and increasing confidence for practice (2/6, 33%).

**Table 1:** Participant perceptions of the interactive clinical avatar simulation.

Statement number	Description	Agreement score, median (IQR)
1	Simulation provided a realistic patient simulation.	4.5 (1)
2	When completing the simulations, I felt as if I were the pharmacist caring for this patient.	5.0 (1.75)
3	When completing the simulations, I felt I had to make the same decisions as a pharmacist would in real life.	4.5 (1)
4	Simulations were interesting.	4.5 (1)
5	Simulations were enjoyable.	4.5 (1)
6	Difficulty of the simulations was appropriate for my level of training.	4.0 (0.75)
7	Feedback I received was adequate for my needs.	5.0 (1)
8	Objectives for the simulations were clear and easy to understand.	4.0 (0.75)
9	I was able to access the simulations at my convenience.	5.0 (1)
10	Simulations helped develop my clinical reasoning skills.	4.5 (1)
11	Simulations helped develop my problem-solving and decision-making skills.	4.0 (1)
12	Simulations have helped me put theory into practice.	4.5 (1)
13	I am confident I am developing the skills required in practice from the simulations.	4.0 (0.75)
14	I am confident I am gaining the knowledge required in practice from the simulations.	4.5 (1)
15	It is my responsibility to learn what I need to know from the simulations.	5.0 (1)
16	Completing the simulations has improved my confidence for the preregistration exam.	4.5 (1)
17	I feel better prepared to care for real-life patients.	4.0 (0.75)
18	I feel more confident about collaborating with patients and other health care professionals.	4.0 (0)
19	Simulations have increased my confidence about practicing as a pharmacist.	4.0 (0.75)
20	Overall, the experience has enhanced my learning.	4.5 (1)

### Qualitative Analysis

Key themes emerged from content analysis of the open-ended questions on the questionnaire regarding use of the case studies as learning tools, use of the case studies in the preregistration training year, limitations of the case studies, and suggestions for improvements of the case studies. Themes and frequencies from the content analysis can be found in Multimedia Appendix 2.

Participants reported the interactive clinical avatar simulation as enjoyable and easy to use as a learning tool. It was described as providing a different format of learning, and the realism and relevance to practice of the simulation promoted experiential learning.

I liked that there was a selection of answers to reply to the patient, more than just a “yes” and “no”...I feel this is how I would communicate with a patient politely, and it made the experience a lot more realistic.

P4, preregistration trainee

One participant specifically commented that the level of feedback was helpful to identify what they did well and how they could improve, while drawing on the repeatable aspect of the simulation.

It helps put learnt knowledge into practice...in a safe and nonpressured environment. I could make a mistake and learn from it; learn how to do a proper, effective consultation without compromising patient care in real life.

P2, preregistration trainee

Participants reported that the interactive clinical avatar could be a useful learning tool in preregistration training; it was primarily thought of as an individual revision aid or a group learning tool. A small number of participants commented on the use of interactive clinical avatar as a tool to help prepare for an Objective Structured Clinical Examination (OSCE).

This case study would fit well in an OSCE scenario...more realistic than a written station and you wouldn’t need to bring in a [standardized patient].

P7, newly qualified pharmacist

Participants reported some limitations of the interactive clinical avatar. The most frequent concern related to the free-text part of the simulation.

At the end of the case study where I had to ask questions. I didn’t realize that this section could also be used to counsel and give advice to the patient. I felt this wasn’t made clear.

P3, newly qualified pharmacist

The free-text entry was also associated with software recognition problems.

It was difficult to enter information as the programme often didn’t recognize these. It became frustrating thinking how to reword things to make the programme recognize them.

P5, newly qualified pharmacist

Four improvements were suggested: providing an example simulation, including a help button or email address for technical issues, including key learning points at the end of the case study, and increasing the size of the question bank (most commonly suggested).

Have a bigger question bank so the simulation can recognize what you are asking and make it easier to progress through the case...it makes you not want to carry on with the consultation.

P9, preregistration trainee

## Discussion

### Principal Findings

The aim of the study was to describe the design and review of three interactive clinical avatar simulations as part of pharmacist preregistration training. Creating the interactive clinical avatar simulations involved multiple reviews and subsequent amendments to ensure content and face validity of the cases. Further measures of concurrent or construct validity were considered and dismissed as not appropriate because of the lack of validated instruments and, therefore, the inability for findings to be correlated with those from another instrument. A sample of preregistration trainees and newly qualified pharmacists reviewed the EHC simulation and reported it to be an effective learning tool, suggesting improvements to increase its usability. The wording, relevance, and difficulty of the interactive clinical avatar were reported as being appropriate with no changes required. The data collection questionnaire was found to be reliable, as indicated by the high Cronbach alpha score.

There has been only one previous study evaluating interactive clinical avatars in preregistration pharmacy training [10], which focused on improving users’ knowledge and confidence of a single community pharmacy service. In contrast to our study, there were few specifics on the simulation design process or validity of the self-rating scale. Previous literature reviews [2,6,7,59,60] concluded the need for further research to be undertaken on the role of virtual patients in postgraduate and continuing pharmacy education. The design of the interactive clinical avatars in this paper goes some way to filling this gap in current research and provides a rationale for a combination of input styles to determine the range of skills and knowledge that can be developed from these simulations [35,44].

This study indicated a high level of satisfaction by preregistration trainees and newly qualified pharmacists with the use of interactive clinical avatars as a learning tool, mirroring the wider literature [12,13,61-65]. Providing engaging and enjoyable learning environments are key components of the learning process [66]. Participants in this study enjoyed completing the EHC simulation, describing it as a novel learning tool that had not been widely used in participants’ undergraduate education. The increasingly digitally native generation of students justifies the creation and evaluation of these more interactive learning tools that may promote self-directed and distance learning, potentially increasing the scope of students who can use them [67].

Participants commented on the realistic design and relevance of the scenarios to real practice, implying high levels of cognitive realism [68]. The interactivity and fidelity of the interactive clinical avatar may have added to users’ immersion in the simulation and their experiential, situated learning; such learning has previously been proposed to occur only with real-life or concrete experiences [69]. Participants reported that the interactive clinical avatar provided them with a means to explore higher order skills such as clinical reasoning. In contrast, the development of more simple skills (such as problem solving or decision making) were not reported as freely, especially by newly qualified pharmacists. This is not necessarily an issue, as previous studies have found that lower order skills can be acquired through straightforward rote learning, but it remains essential to ensure that any learning tool is created with the level of learners and intended learning outcomes in mind [70]. Although virtual patients have been established as tools to develop health care professionals’ clinical reasoning skills [2,8,28,71,72], little work has been conducted within the pharmacy profession or evaluating interactive clinical avatars specifically. Findings from this study also mirror the wider literature regarding knowledge development post virtual patient use, with preregistration trainees and newly qualified pharmacists reporting the development of knowledge required for future practice after completing the interactive clinical avatar simulation [10,13,73-79]. Resources that allow users to immerse themselves in a scenario and behave as they would in real practice are extremely beneficial in aiding the development of knowledge, skills, and confidence and may bridge the gap between theory and practice in preregistration training [80].

Learning tools must reflect the level of education that individuals are at and allow a spirality of content to ensure new learning is linked to previous learning and students’ competence can develop in line with the difficulty of task [81]. Interactive clinical avatars may add little value when learners are at the lower levels of Bloom’s taxonomy [82]; they should instead be used when knowledge is combined with skills and applied in problem-solving scenarios or when direct patient contact is not possible. This may explain why preregistration trainees were more agreeable to the simulations allowing an opportunity to put theory into practice, developing skills, improving confidence for real-life practice, and collaborating with patients and health care professionals; newly qualified pharmacists may have had this experience in real-life practice. This demonstrates that interactive clinical avatars are able to help promote progression through the levels of Miller’s triangle, as per other simulation-based learning [83].

The integration of a decision tree into simulation design allows users to dynamically control the case study and assures outcomes are directly determined by user input to encourage a greater sense of realism and responsibility. Interactive clinical avatar simulations provide a safe environment for users to repeatedly practice, change the simulation outcome, receive the appropriate feedback, and reflectively learn. This was noted as being particularly beneficial in preregistration training, as it would allow individuals to learn from experiences they may not otherwise have.

### Strengths and Limitations

All interactive clinical avatar case studies were reviewed by the research team and qualified pharmacists to increase their validity. The EHC simulation was also reviewed by a sample of preregistration trainees and newly qualified pharmacists. The sample size of 10 may be considered small and findings due to chance, but the purpose was to review the design of the interactive clinical avatar simulation and data collection tool, for which data saturation was reached. All comments received were useful regarding the interactive clinical avatar design—in particular, improvements suggested related to features consistent for all three case studies (eg, providing an example interactive clinical avatar simulation, signposting for help, and listing key points relating to the topic). The improvements regarding the question bank and recognition of the avatar can also be translated across the interactive clinical avatar simulations where free-text input is used.

Perceptions of the interactive clinical avatar were obtained via a questionnaire. The self-reporting aspect did not show whether participants’ knowledge or skills related to completing an EHC consultation did actually improve, only their thoughts of such. The primary focus of the questionnaire was to obtain participant perceptions relating to the avatar simulation rather than evaluating its ability to promote knowledge or skill development; this will be part of a larger study.

### Conclusions

The design and creation of the interactive clinical avatar simulations was successful. The multistep review process ensured validity and reliability of the simulations and data collection questionnaire. The in-depth explanation of the design process and provision of a questionnaire may help widen the use and evaluation of interactive clinical avatars or other simulation tools.

The interactive clinical avatars were received favorably as a novel learning tool that was reported to promote experiential learning and allow individuals to feel like they were making real-life decisions, developing transferable knowledge and skills. Improvements were suggested to increase the use of the interactive clinical avatars, and amendments will be made for further, larger scale evaluations. The use of interactive clinical avatars as learning tools can provide potential benefits to many health care training courses because they provide standardized simulations that allow active learning and reflection.

## Appendix

Multimedia Appendix 1 Emergency hormonal contraception interactive clinical avatar demonstration.

Multimedia Appendix 2 Content analysis themes and frequencies.

## Abbreviations

3D: 3-dimensional
EHC: emergency hormonal contraception
OSCE: Objective Structured Clinical Examination
