# Supplementary material for: Interactive Clinical Avatar Use in Pharmacist Preregistration Training: Design and Review
Source: J Med Internet Res. 2020 Nov 6;22(11):e17146. doi: 10.2196/17146 (PMC7679212; doi:10.2196/17146)
Supplement: Multimedia Appendix 2 [file jmir_v22i11e17146_app2.pdf]

Theme	Number of participants	
	Pre-registration trainees	NQPs
**Use of the case studies as learning tools**		
Novelty	2	3
Realism	3	4
Experiential Learning	4	4
Feedback	4	2
**Integration of the learning tools**		
Individual revision aid	2	4
Group learning	3	1
OSCE practice	1	2
**Limitations of the case studies**		
Confusion with case completion	4	5
Recognition aspects	2	4
**Suggestions for improvements**		
Example case	2	1
‘Help’ button	2	3
Key points	1	3
Question bank size	2	5
